# Inhibition of STAT3 by Niclosamide Synergizes with Erlotinib against Head and Neck Cancer

**DOI:** 10.1371/journal.pone.0074670

**Published:** 2013-09-03

**Authors:** Rui Li, Shuo You, Zhongliang Hu, Zhuo G. Chen, Gabriel L. Sica, Fadlo R. Khuri, Walter J. Curran, Dong M. Shin, Xingming Deng

**Affiliations:** 1 Department of Radiation Oncology, Emory University School of Medicine and Winship Cancer Institute, Emory University, Atlanta, Georgia, United States of America; 2 Department of Hematology and Medical Oncology, Emory University School of Medicine and Winship Cancer Institute, Emory University, Atlanta, Georgia, United States of America; 3 Department of Pathology, Emory University School of Medicine and Winship Cancer Institute, Emory University, Atlanta, Georgia, United States of America; University of California Irvine, United States of America

## Abstract

Epidermal growth factor receptor (EGFR) is extensively expressed in head and neck cancer. However, EGFR-targeted therapy has only modest efficacy in head and neck cancer, through mechanisms that are not fully understood. Here, we found that inhibition of EGFR by erlotinib stimulated phosphorylation and activation of STAT3 leading to increased Bcl2/Bcl-XL expression in head and neck cancer cells, which may dampen the therapeutic efficacy of erlotinib against head and neck cancer. Erlotinib-enhanced STAT3 phosphorylation results, at least in part, from suppression of its physiological phosphatase, PTPMeg2. Specific knockdown of STAT3 by RNA interference significantly sensitized head and neck cancer cells to erlotinib treatment. Pharmacological inhibition of STAT3 by niclosamide not only blocked erlotinib-stimulated STAT3 phosphorylation but also synergistically repressed head and neck cancer growth *in vitro* and *in vivo*. Combined inhibition of EGFR and STAT3 by erlotinib and niclosamide more effectively induced apoptosis in tumor tissues without toxicity for normal tissues. Based on our findings, treatment with erlotinib combined with niclosamide may offer an effective therapeutic approach to improve the prognosis of head and neck cancer.

## Introduction

Head and neck cancers consistently rank among the six most frequently diagnosed cancers in the world [1]. Over 90% of head and neck cancers are squamous cell carcinomas of the upper aerodigestive tract, including the oral cavity, pharynx, larynx, and paranasal sinuses (1). Squamous cell carcinoma of the head and neck (SCCHN) constitutes an estimated 2.5% of cancer diagnoses in the United States, with 41,380 new cases diagnosed and an estimated 7,890 deaths in 2013 [2]. Despite advances in conventional therapies, including surgery, radiation and chemotherapy, the overall survival rate for SCCHN has not been significantly improved in the past decades . Epidermal growth factor receptor (EGFR) is widely expressed in SCCHN and has been used as a critical target for treatment of this disease [3]. Erlotinib, an EGFR tyrosine kinase inhibitor (TKI), has been used for the treatment of SCCHN but its efficacy is modest [5]. The mechanism accounting for the limited efficacy of erlotinib in head and neck cancer therapy is not fully understood. It is possible that inhibition of EGFR by erlotinib may induce activation of some survival signaling pathway(s) that dampens the efficacy of erlotinib against SCCHN.

STAT3, a member of the STAT family of transcription factors, is activated in several cancers, and has recently been validated as an attractive therapeutic target in cancer therapy, including head and neck cancer [6–10]. In addition, SCCHN specimens also have higher levels of STAT3 than normal tissue [11]. Latent cytoplasmic STAT3 becomes activated through phosphorylation at residue Tyr705 by Janus associated kinase (JAK) or growth factor receptor-associated tyrosine kinase (Src) [12]. Phosphorylated STAT3 dimerizes through a reciprocal Src homology 2-phospho-tyrosine interaction and accumulates in the nucleus, where it activates the transcription of a wide array of genes, including Bcl2/Bcl-XL, cyclin D1, c-Myc, etc [13,14]. In addition to STAT3 kinases (i.e. JAK), STAT3 phosphorylation is also tightly regulated by a process of dephosphorylation, which is mediated by the protein tyrosine phosphatase PTPMeg2 [15]. PTPMeg2 has recently been identified as a new physiologic STAT3 phosphatase that directly dephosphorylates STAT3 at the Tyr705 residue [15]. Intratumoral administration of STAT3 decoy inhibited the growth of SCCHN xenograft tumors *in vivo* [16], suggesting that STAT3 is a potential therapeutic target for SCCHN. Despite the promise of a number of rational approaches to target STAT3 function, no small molecule STAT3 inhibitor appears to be ready for clinical development so far [17].

Niclosamide has recently been reported as a potent STAT3 inhibitor against cancer cells [18]. In this report, we show that erlotinib enhances STAT3 phosphorylation by downregulation of its phosphatase PTPMeg2 leading to elevated levels of Bcl2/Bcl-XL, which reduces sensitivity of SCCHN to erlotinib treatment. Niclosamide, as a STAT3 inhibitor, can block erlotinib-induced phosphorylation of STAT3 leading to synergistic suppression of head and neck cancer.

## Materials and Methods

### Materials

Niclosamide was purchased from Sigma-Aldrich (St. Louis, MO, USA). Erlotinib was obtained from LC Laboratories (Woburn, MA). Phospho-EGFR (Tyr1068), phospho-STAT3 (Tyr705), phospho-JAK2 (Tyr1007/1008), active caspase 3 and survivin antibodies were purchased from Cell Signaling Technology (Beverly, MA). β-actin and Mcl-1 antibodies, PTPMeg2 shRNA and its control shRNA were purchased from Santa Cruz Biotechnology (Santa Cruz, CA). PTPMeg2 antibody was obtained from R&D systems (R&D systems, MN). Bcl2 was obtained from Calbiochem (Darmstadt, Germany). Bcl-XL was purchased from Epitomics, Inc. (Burlingame, CA). QD605 goat anti-rabbit IgG conjugate (red), QD705 goat anti-mouse IgG conjugate (green) and ProLong® Gold antifade reagent with 4', 6-diamidino-2-phenylindole (DAPI) were purchased from Invitrogen Life Technologies Inc (Carlsbad, CA). All other reagents used were obtained from commercial sources unless otherwise stated.

### Cell lines and cell culture

Human SCCHN cell lines Tu212 and Tu686 were established from primary HNSCCs and maintained in DMEM/F12 (1:1) medium with 10% fetal bovine serum as described previously [19]. These cell lines were employed for the described experiments without further authentication.

### Preparation of cell lysates and Western blot

Cells were washed with cold PBS and resuspended in ice-cold EBC buffer (0.5% Nonidet P-40, 50mM Tris, pH 7.6, 120 mM NaCl, 1 mM EDTA, and 1 mM- β-mercaptoethanol) containing protease inhibitor mixture set I. Following cell lysis by sonication and centrifugation at 14,000 x g for 15 min at 4 °C, the resulting supernatant was collected as the total cell lysate. Protein expression was analyzed by Western blot as previously described [20].

### Quantitative reverse transcription PCR (RT-PCR)

For quantitative RT-PCR, total RNA was purified and reverse transcribed with random hexamers and SuperScript III (Invitrogen). Amplification was carried out using 2 × SYBR green PCR mix (Bio-Rad, CA) by ABI 7500 real-time PCR system (Applied Biosystems) as described [21,22]. Specific primers: for human Bcl2: forward, 5′-GGT GGA GGA GCT CTT CAG G-3′ and reverse, 5′- ATA GTT CCA CAA AGG CAT CC-3′; for human Bcl-XL: forward, 5′- ATA GTT CCA CAA AGG CAT CC -3′ and reverse, 5′- TGG GAT GTC AGG TCA CTG AA-3′; and for β-actin: forward, 5′- TCA GGA TCC ACG TGC TTG TCA -3′; reverse, 5′- TAC CCT TGG ACC CAG AGG TTC TTT GA -3′. Relative gene expression quantifications were performed according to the comparative Ct method using β-actin as an internal standard. Quantification of gene expression was analyzed with the 7500 v 2.0.5 software program and quantified by the 2-ΔΔCt method. Data represent the mean ± SD of three independent experiments.

### RNA Interference

Lentiviral pSIH1-puro-STAT3 shRNA and pSIH1-puro-control shRNA were obtained from Addgene (Cambridge, MA). The control shRNA plasmid-A encodes a scrambled shRNA sequence that will not lead to the specific degradation of any cellular message. Control shRNA hairpin sequence: CCT AAG GTT AAG TCG CCC TCG CTC GAG CGA GGG CGA CTT AAC CTT AGG. STAT3 shRNA hairpin sequence: GAT CCG CAT CTG CCT AGA TCG GCT ATT CAA GAG ATA GCC GAT CTA GGC AGA TGT TTT TTG. PTPMeg2 shRNA and its control shRNA were purchased from Santa Cruz Biotechnology (Santa Cruz, CA). For pseudovirus production, STAT3 shRNA or PTPMeg2 shRNA was cotransfected into 293FT cells with the lentivector packaging plasmid mixture (System Biosciences, CA) using NanoJuice transfection kit (EMD Chemical, Inc.) as described [23]. After 48h, the virus-containing media were harvested by centrifugation at 20,000 × g. Cells were infected with the virus-containing media in the presence of polybrene (8µg/ml) for 24h following which stable positive clones were selected using 1µg/ml puromycin.

### Sulforhodamine B (SRB) assay

The inhibitory effects of erlotinib and niclosamide on cell growth were assessed using the sulforhodamine B (SRB) assay as described [24]. Cells were cultured in triplicate wells using 96-well plates. After overnight growth, cells were treated with erlotinib, niclosamide or the combination for 48h. The surviving cell fraction was determined as described [24].

### Head and neck cancer xenografts and treatments

The Institutional Animal Care and Use Committee of Emory University approved the protocol for animal experiments. Six-week-old Nu/Nu nude mice were purchased from Harlan and housed under pathogen-free conditions in microisolator cages. Xenografts were raised by injecting 5 ×10^6^ of Tu212 cells in a balanced salt solution into subcutaneous tissue over the flank region of nude mice. Tumors were allowed to grow to an average volume of 250 mm^3^ prior to initiation of therapy as described [25]. Tumor-bearing mice were randomized into four treatment groups (n = 8 per group) as follows: (1) vehicle control (0.5% DMSO, 100µl/d i.p.); (2) erlotinib (40mg/kg/d i.p.); (3) niclosamide (20mg/kg/d i.p.); (4) erlotinib (40mg/kg/d) + niclosamide (20mg/kg/d). Tumor volume was assessed by caliper measurements once every two days and calculated with the formula: V=(LxW^2^)/2 (L: length; W: width) as described [26]. After 14 consecutive days of treatment, mice were sacrificed by inhaled CO_2_. Harvested tumors were used for further analysis.

### Immunohistochemistry (IHC) analysis

IHC staining of tumor tissue using rabbit anti-human active caspase 3 antibody was performed as described [25]. Briefly, harvested tumors were embedded in paraffin and cut into 4-µm sections. Staining was performed using the R.T.U. Vectastain kit following the manufacturer’s standard protocol (Vector Laboratories, Burlingame, CA). The tissue slides were blocked with 2.5% normal horse serum for 10 min. Samples were then incubated with rabbit anti-human active caspase 3 antibody (dilution 1:50), overnight at 4°C. After washing, the tissue slides were incubated with anti-rabbit IgG HRP secondary antibody for 10 minutes. The slides were stained with 3,3’-diaminobenzidine (DAB) (Vector Laboratories) and counterstained with hematoxylin (Vector Laboratories), dehydrated, treated with xylene, and mounted. All slides were examined and representative pictures were taken using an Olympus BX41 microscope (Olympus, America, Melville, NY). Active caspase-positive cells in tumor tissues were scored at 400 × magnification. The average number of positive cells per 0.0625 mm^2^ area was determined from three separate fields in each of three independent tumor samples as described [25].

### Quantum dot-based immunohistoﬂuorescence (QD-IHF) and its signal quantification

QD-IHF for measurement of protein expression in tumor tissues was performed as described previously [27–29]. Briefly, harvested tumors were embedded in paraffin and cut into 4-µm sections. After deparaffinization and rehydration, antigen retrieval was performed by heating with citrate buffer (10 mmol/L, pH 6.0) in a microwave for 10 min. The tissue slides were blocked with 2.5% normal horse serum for 10 min before the primary antibody incubation. Rabbit anti-human pEGFR and mouse anti-human p-STAT3 antibodies were mixed at 1:50 dilution in 1×PBS containing 2.5% horse serum. Normal rabbit IgG was used as a negative control. Tissue sections were incubated with a mixed solution of p-EGFR and p-STAT3 antibodies overnight at 4°C. After washing with 1×PBS three times, QD705 goat anti-mouse IgG conjugate (green) and QD605 goat anti-rabbit IgG conjugate (red) secondary antibodies were added to the slides with further incubation for 1h at 37°C. The slides were washed three times with 1×PBS, counterstained with DAPI, mounted and stored at 4^o^C under dark conditions. QD imaging and quantification procedures were performed as described previously [29]. The Nuance^TM^ fluorescence microscope system (CRi consolidated with Caliper, a PerkinElmer company, Hopkinton, MA) was used for quantification of the QD-IHF signals. All cubed image files were collected from tumor tissue slides at 10 nm wavelength intervals from 420–720 nm, with an auto exposure time per wavelength interval at 200 ~ 400x magnification. Taking the cube with a long wavelength band pass filter allowed transmission of all emission wavelengths above 420 nm. Both separated and combined QD images were obtained by unmixing the image cube based on establishing the QD spectral library. For each tissue slide, 10 cubes were taken. The background signal was removed for accurate quantification of the QD signals. The average of each QD signal was obtained by selecting tumor areas on each cube for quantification by Nuance imaging software (Caliper/PerkinElmer). An average reading from the 10 cubes was obtained as a total average signal count of each tissue slide for both pEGFR and pSTAT3 signals.

### Mouse blood analysis

Whole blood (250µL) was collected in EDTA-coated tubes via cardiac puncture of anesthetized mice for hematology studies. Specimens were analyzed for white blood cells (WBC), red blood cells (RBC), platelets (PLT), alanine aminotransferase (ALT), aspartate aminotransferase (AST) and blood urea nitrogen (BUN) in the Clinical Pathology Laboratory at the University of Georgia (Athens, GA).

### Statistical analysis

Significant differences between two groups were analyzed using two-sided unpaired Student’s t-test and p value < 0.05 was considered statistically significant. Statistical analysis was performed with Graphpad InStat 3 software (San Diego, CA) [30].

### Analysis of combination index (CI) value

CI value for drug synergy was calculated using the CompuSyn software (Combo-Syn, Inc., Paramus, NJ) as described [31].

## Results

### Inhibition of EGFR by erlotinib downregulates PTPMeg2 leading to enhanced STAT3 phosphorylation

**Figure 1:**
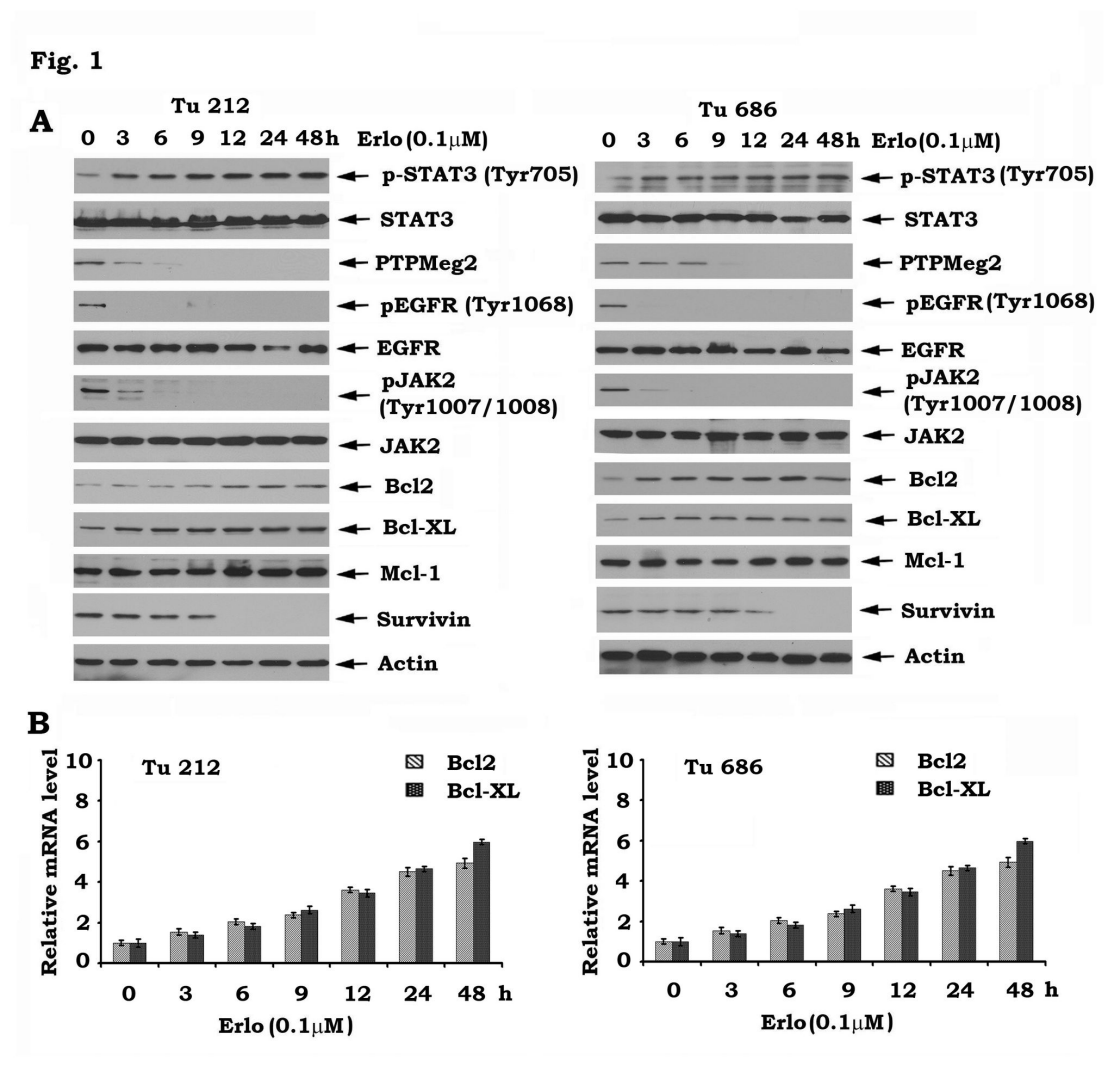
Inhibition of EGFR by erlotinib downregulates PTPMeg2 leading to enhanced phosphorylation of STAT3 in head and neck cancer cells. *A*, Tu212 and Tu686 cells were treated with erlotinib (0.1µM) for various times. Levels of various proteins (pSTAT3, PTPMeg2, pEGFR, Bcl2, Bcl-XL, etc.) were analyzed by Western blot. *B*, Tu212 and Tu686 cells were treated with erlotinib (0.1µM) for various times. Levels of Bcl2 or Bcl-XL mRNA were analyzed by RT-PCR.

It has been recently reported that inhibition of EGFR by erlotinib activates the STAT3/Bcl2/Bcl-XL survival signaling pathway in human lung cancer [32]. To test whether this effect of erlotinib occurs in head and neck cancer cells, Tu212 and Tu686 cells were treated with erlotinib (0.1µM) for various times. Results indicated that erlotinib reduced EGFR phosphorylation in association with enhanced Tyr705 STAT3 phosphorylation, increased levels of Bcl2/Bcl-XL and decreased levels of survivin (Figure 1A). STAT3 is a physiologic transcription factor of Bcl2 and Bcl-XL [33–35]. Increased levels of Bcl2 and Bcl-XL mRNA were also observed in Tu212 and Tu686 cells after erlotinib treatment (Figure 1B), indicating that erlotinib-activated STAT3 positively regulates Bcl2/Bcl-XL but not survivin at the transcriptional stage. To demonstrate how erlotinib stimulates STAT3 phosphorylation, the effects of erlotinib on STAT3 kinase (i.e. JAK2) and phosphatase (i.e. PTPMeg2) were assessed. Intriguingly, erlotinib not only inhibited JAK2 phosphorylation but also significantly reduced PTPMeg2 expression level in head and neck cancer cells (Figure 1A). These findings indicate that erlotinib-mediated STAT3 phosphorylation and activation may result from inhibition of the STAT3 phosphatase PTPMeg2.

**Figure 2:**
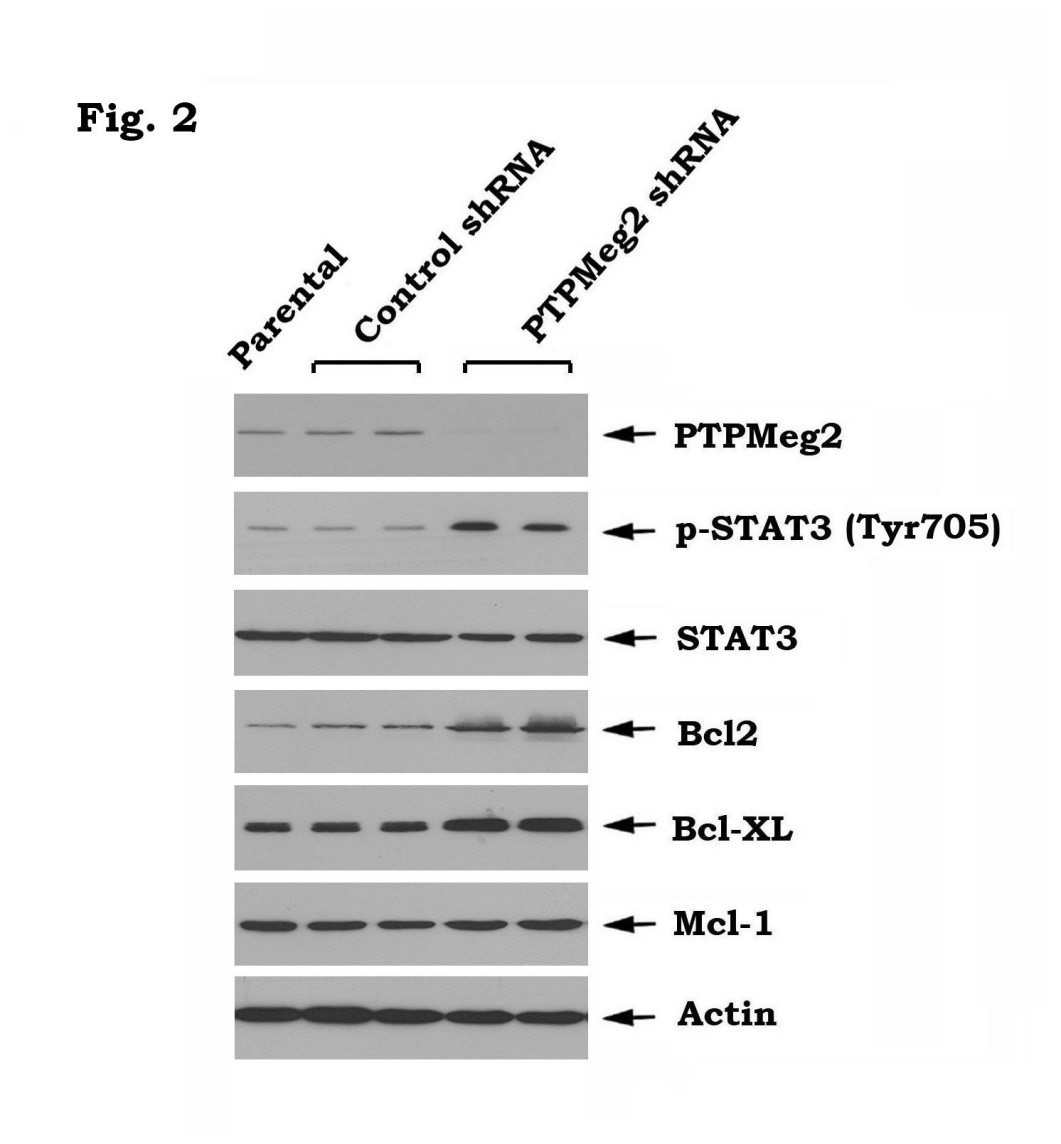
Knockdown of PTPMeg2 leads to upregulation of pSTAT3 and Bcl2/Bcl-XL in Tu212 cells. PTPMeg2 shRNA or control shRNA was transfected into Tu212 cells. Expression levels of pSTAT3, Bcl-XL, Bcl2 and Mcl-1 were analyzed by Western blot.

Importantly, specific knockdown of PTPMeg2 also led to increased STAT3 phosphorylation and elevated levels of Bcl2 and Bcl-XL in head and neck cancer cells (Figure 2), which further supports the role of PTPMeg2 as a physiologic STAT3 phosphatase, as recently reported [15].

### Disruption of STAT3 sensitizes head and neck cancer cells to erlotinib

**Figure 3:**
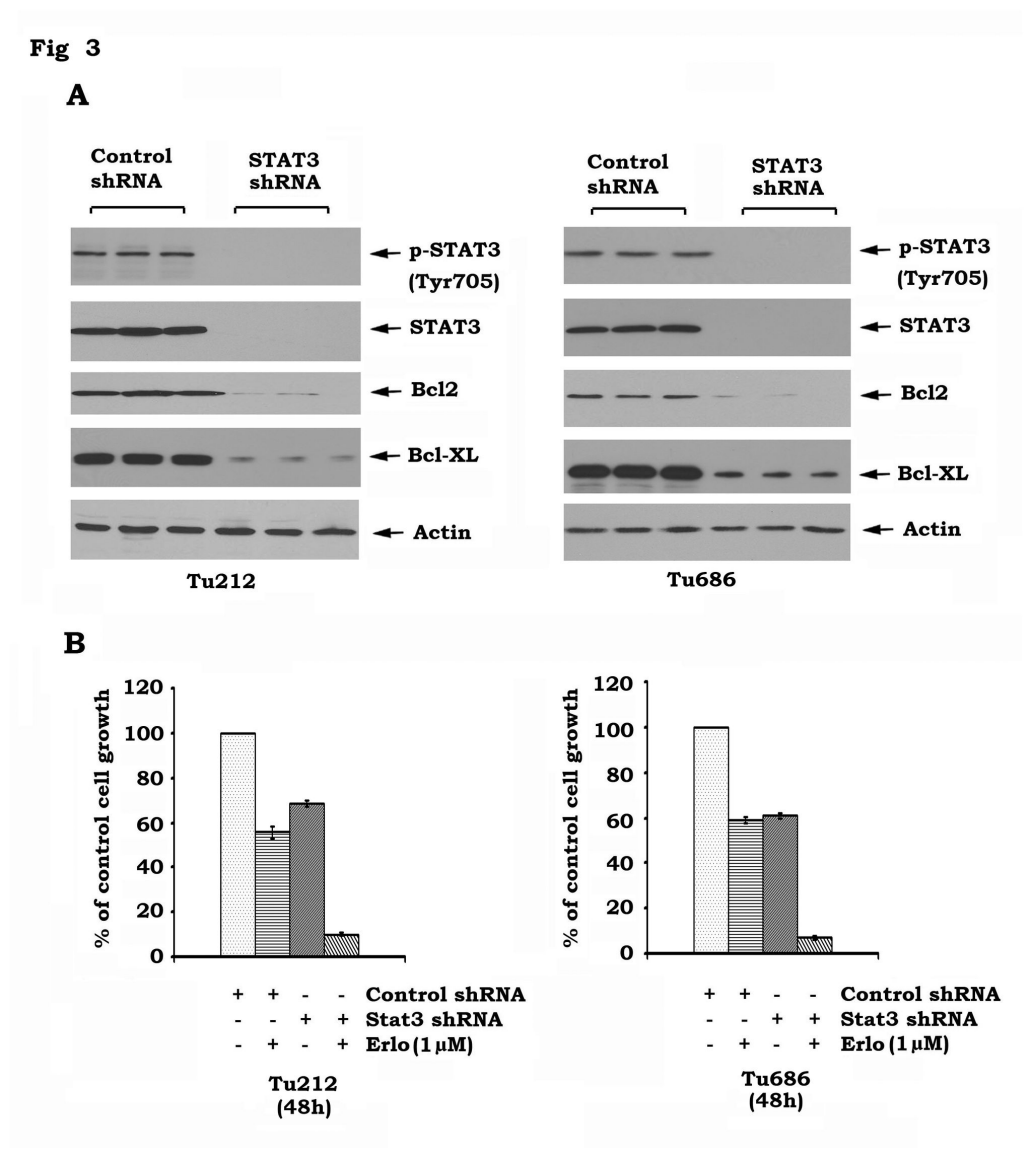
Specific depletion of STAT3 sensitizes head and neck cancer cells to erlotinib. *A*, STAT3 shRNA or control shRNA was transfected into Tu212 and Tu686 cells. Expression levels of STAT3, Bcl-XL and Bcl2 were analyzed by Western blot. *B*, Tu212 and Tu686 cells expressing STAT3 shRNA or control shRNA were treated with erlotinib (1µM) for 48h. Cell growth was determined by SRB assays.

To test whether erlotinib-mediated STAT3 activation negatively affects erlotinib activity against head and neck cancer, STAT3 was knocked down from Tu212 and Tu686 cells using STAT3 shRNA (Figure 3A). Silencing of STAT3 not only downregulates Bcl2 and Bcl-XL (Figure 3A) but also significantly sensitizes head and neck cancer cells to erlotinib (Figure 3B), suggesting that targeting STAT3 may have great potential to improve the efficacy of erlotinib against head and neck cancer.

Niclosamide blocks erlotinib-induced STAT3 phosphorylation and synergizes with erlotinib in the suppression of head and neck cancer cell growth

**Figure 4:**
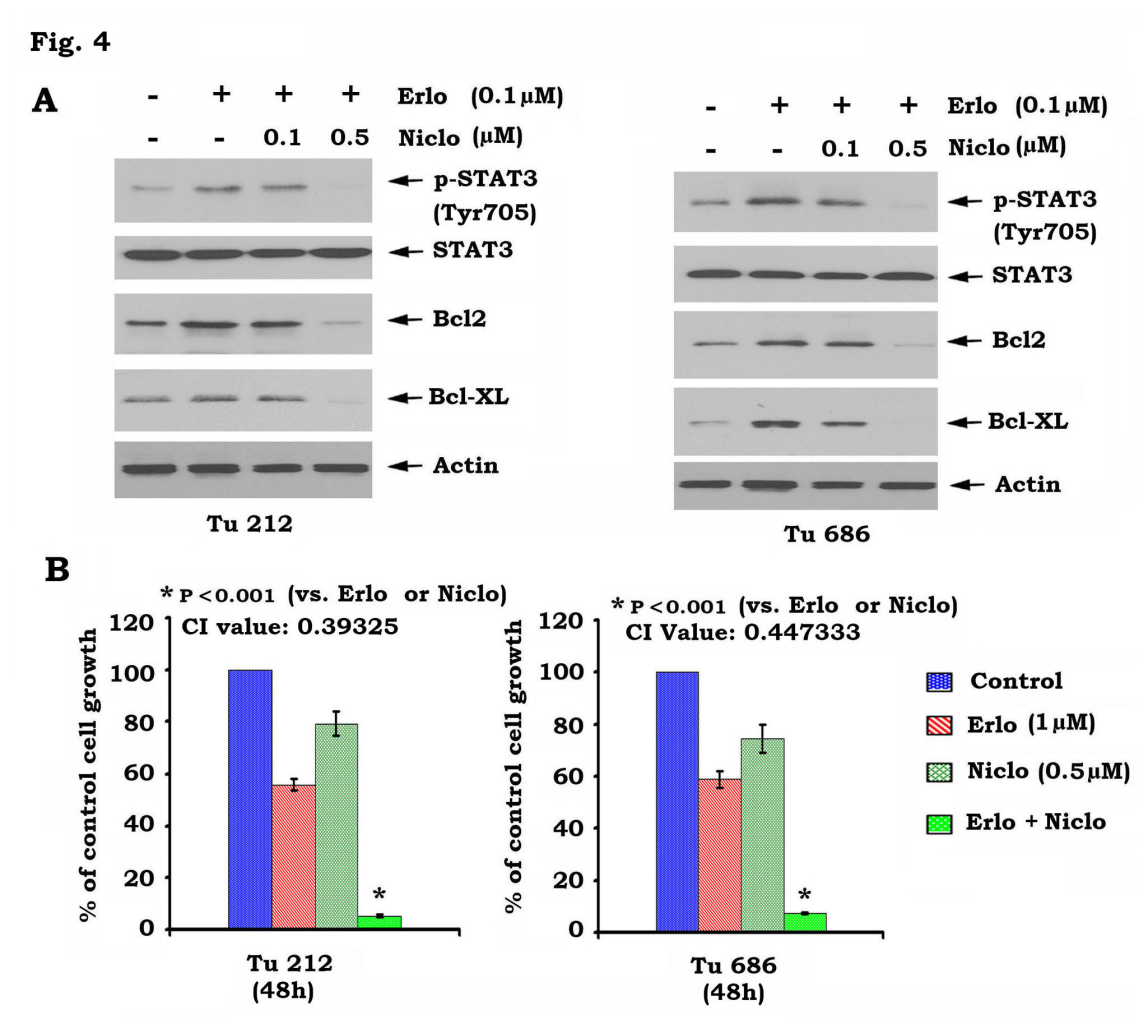
Inhibition of STAT3 by niclosamide blocks erlotinib-induced STAT3 phosphorylation and sensitizes head and neck cancer cells to erlotinib. *A*, Tu212 and Tu686 cells were treated with erlotinib in the absence or presence of increasing concentrations of niclosamide for 48h. pSTAT3, Bcl2 and Bcl-XL were analyzed by Western Blot. *B*, Tu212 and Tu686 cells were treated with erlonitib, niclosamide or their combination. After 48 h, cell growth was determined by SRB assays. Combination index (CI) values were calculated as described in “Methods.

Niclosamide has recently been identified as a potent STAT3 inhibitor [18]. To test whether pharmacological disruption of STAT3 activity sensitizes head and neck cancer cells to erlotinib, Tu212 and Tu686 cells were treated with erlotinib in the absence or presence of increasing concentrations of niclosamide. Western blot analysis showed that niclosamide inhibited erlotinib-induced STAT3 phosphorylation and downregulated Bcl2/Bcl-XL in a dose-dependent manner (Figure 4A). Importantly, niclosamide in combination with erlotinib significantly augmented growth inhibition of head and neck cancer cells (i.e. Tu212 and Tu686) (Figure 4B). To more accurately analyze the degree of synergy between niclosamide and erlotinib, combination index (CI) values were calculated as described in “Methods”. The CI values were 0.39325 for Tu212 and 0.447333 for Tu686 cells, respectively. The CI values of less than 1 indicate that niclosamide and erlotinib exhibit synergistic growth inhibition of head and neck cancer cells.

Niclosamide and erlotinib synergistically inhibit the growth of head and neck cancer animal xenografts

**Figure 5:**
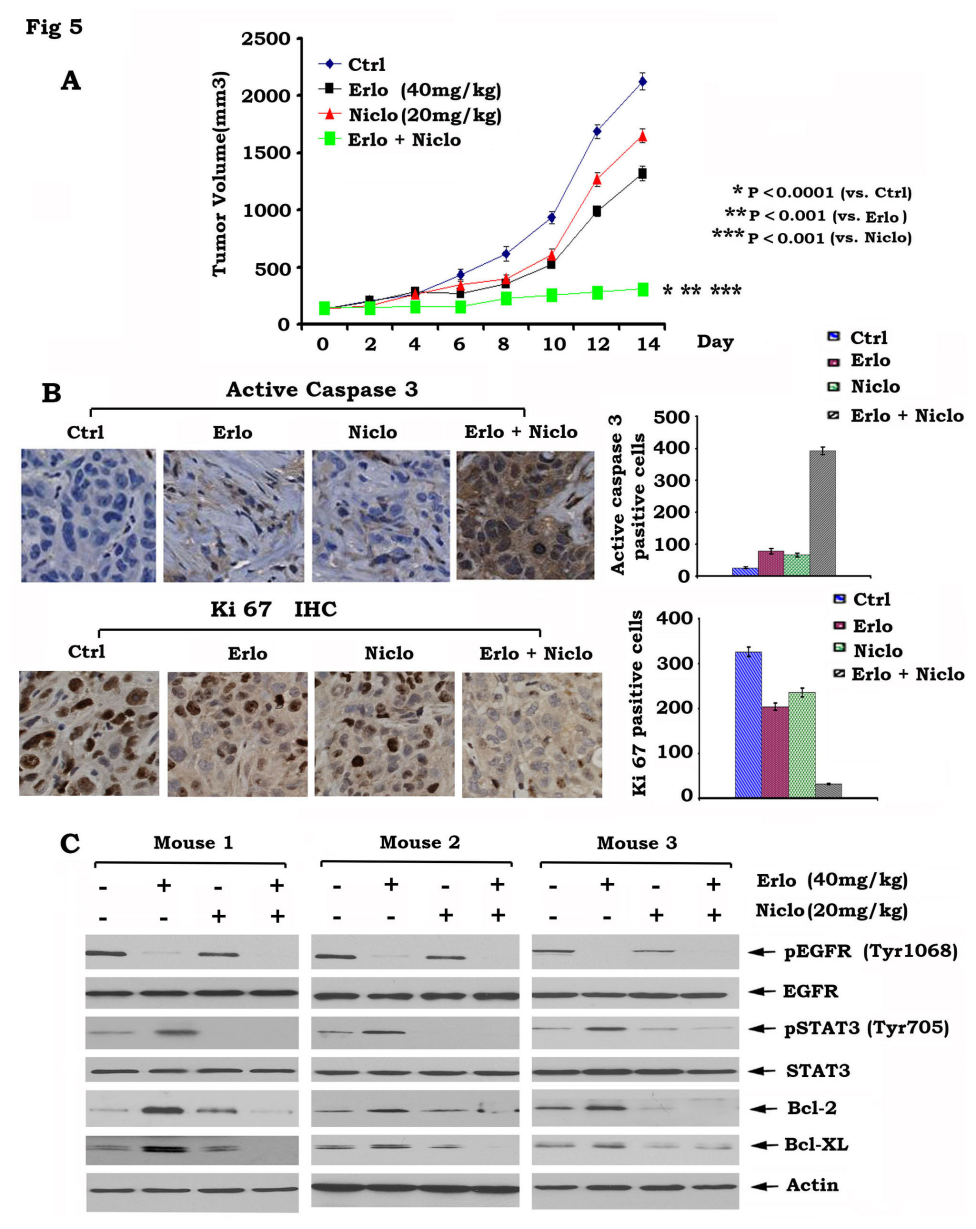
Combination of erlotinib and niclosamide synergistically represses head and neck cancer growth *in vivo*. *A*, Mice bearing Tu212 xenografts were treated with vehicle control, erlotinib (Erlo, 40mg/kg/d), niclosamide (Niclo, 20mg/kg/d) or their combination for 14 days. Each group included 8 mice. Tumor volume was measured once every 2 days. After 14 days, the mice were sacrificed and the tumors were removed and analyzed. Representative tumor pictures were taken. *B*, Active caspase 3 and Ki 67 were analyzed in tumor tissues at the end of experiments by IHC staining and quantified as described in “Methods”. *C*, Expression levels of pEGFR, pSTAT3, Bcl2 and Bcl-XL in tumor tissues from various treatment groups were analyzed by Western blot.

Since niclosamide and erlotinib play a synergistic role against head and neck cancer *in vitro* (Figure 4), it is interesting to examine this synergistic effect *in vivo*. First, head and neck cancer xenografts were generated using Tu212 cells. Then, mice bearing Tu212 head and neck cancer xenografts were treated with erlotinib (40mg/kg/d), niclosamide (20mg/kg/d) alone or in combination for two weeks. Results showed that both erlotinib and niclosamide alone had modest efficacy against the xenograft tumors. However, the combination of erlotinib and niclosamide repressed xenograft tumor formation significantly more efficiently than either single agent alone *in vivo* (p < 0.01) (Figure 5A). These data suggest that niclosamide and erlotinib have strong synergism in the treatment of head and neck cancer.

We measured apoptosis by analysis of active caspase 3 in the tumor tissues by IHC staining as previously described [25]. The combination of erlotinib and niclosamide significantly increased active caspase 3 positive cells in association with decreased Ki-67 positive cells in head and neck tumor tissues (Figure 5B). Protein analysis of tumor tissue lysates from three mice in each treatment group indicated that niclosamide blocks erlotinib-stimulated STAT3 phosphorylation leading to decreased Bcl2 and Bcl-XL levels (Figure 5C). These data suggest the molecular mechanism by which niclosamide and erlotinib exhibit synergism against head and neck cancer growth *in vivo*.

**Figure 6:**
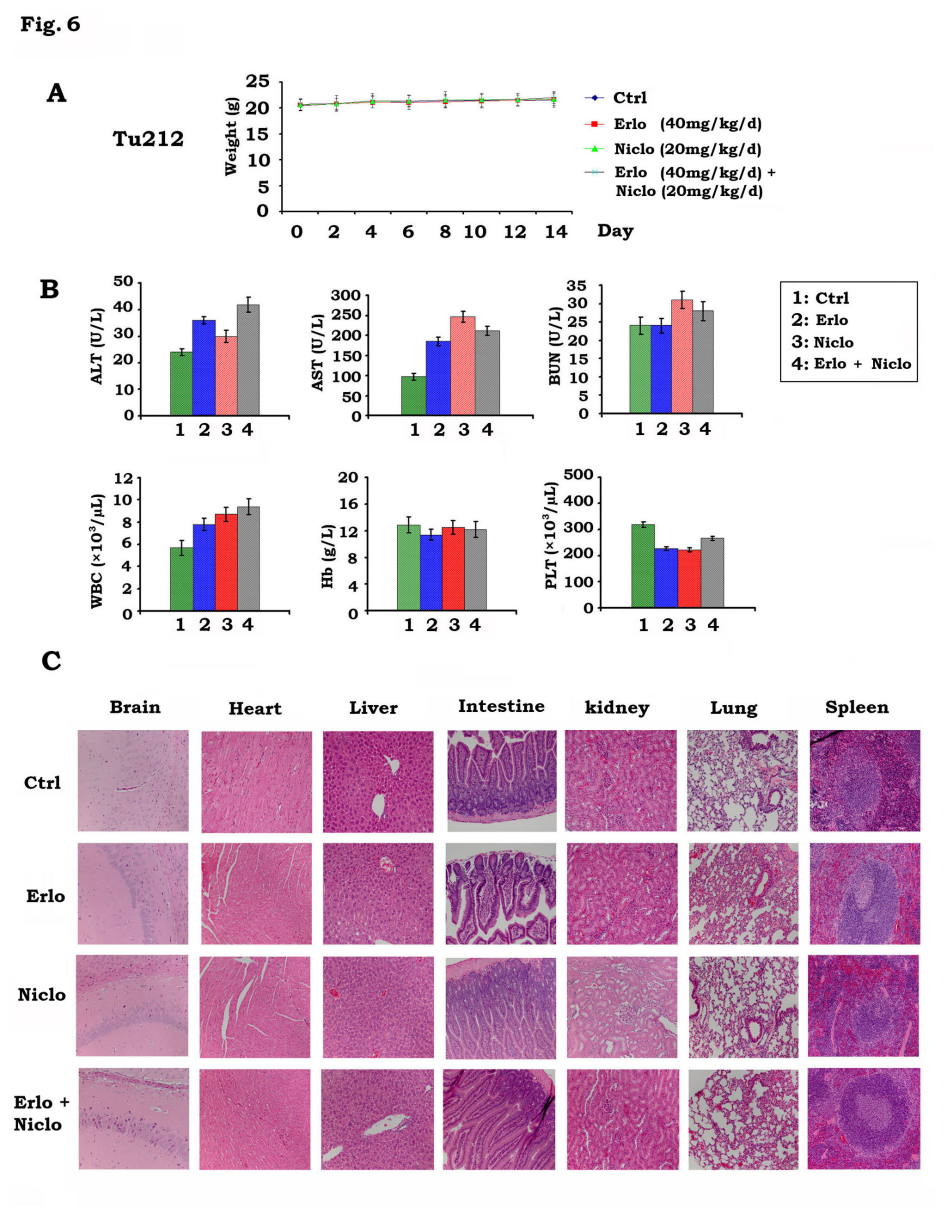
Toxicity analysis in mice bearing Tu212 xenografts treated with erlotinib and niclosamide. *A*, Body weight of mice with Tu212 xenografts was measured once every other day during treatment with vehicle control, erlotinib (Erlo, 40mg/kg/d), niclosamide (Niclo, 20mg/kg/d) or their combination. *B*, Blood analysis of mice after various treatments for 14 days. C, H & E histology of various organs after treatments.

Importantly, treatments were well tolerated without weight loss during treatment (Figure 6A). There were no observable alterations in vital organ functions as reflected by the results of liver, kidney, and bone marrow function tests (ALT, AST and BUN, WBC, Hb and platelet) (Figure 6B). Histopathology of harvested normal tissues (brain, heart, lung, liver, spleen, kidney and intestine) revealed no evidence of toxicity in normal tissue (Figure 6C).

Analysis of pEGFR and pSTAT3 in tumor tissues by quantum dot (QD)-based immunohistofluorescence (QD-IHF)

**Figure 7:**
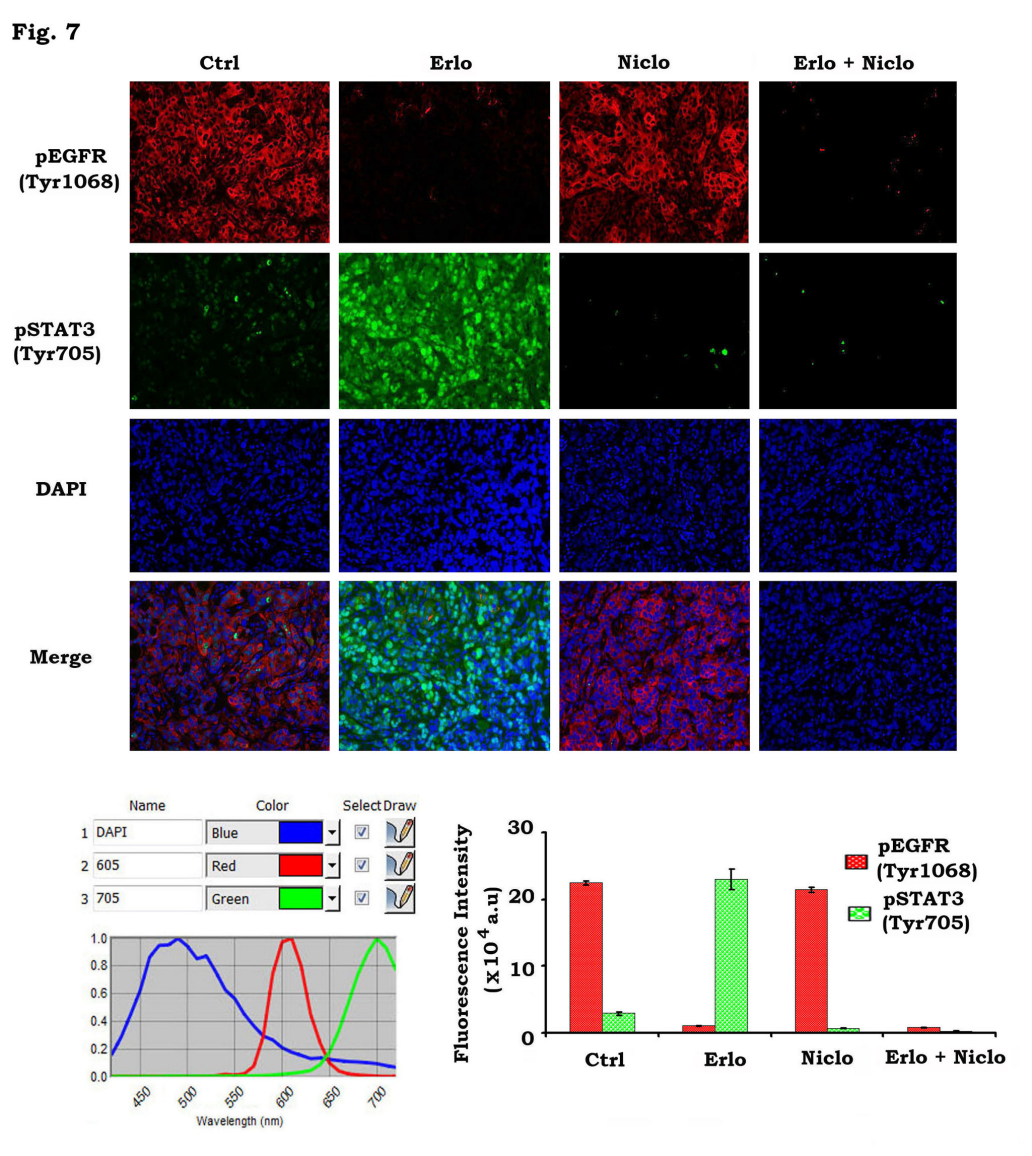
QD-IHF analysis of pEGFR and pSTAT3 in tumor tissues. *A* and *B*, Tu212 xenografts were treated with vehicle control, erlotinib (Erlo, 40mg/kg/d), niclosamide (Niclo, 20mg/kg/d) or their combination for 14 days. pEGFR and pSTAT3 were analyzed in tumor tissues at the end of experiments by QD-IHF and quantified as described in “Methods”.

The QD-IHF technique has a significant advantage in allowing for the quantification of several biomarkers simultaneously on the same tissue slide [27–29,36]. Levels of pEGFR and pSTAT3 were simultaneously analyzed by QD-IHF using primary antibodies and QD-conjugated secondary antibodies with two different emission wavelengths (i.e. QD705 (green) and QD605 (red)). Both separated and combined QD images were obtained after determining the QD spectral library and unmixing the cubed image. QD images from tumor tissues showed that pEGFR was localized at the cell membrane and pSTAT3 was located in the nucleus (Figure 7). Decreased pEGFR and increased pSTAT3 levels were observed in head and neck xenograft tumor tissues after treatment of mice with erlotinib (Figure 7). Furthermore, niclosamide completely blocked erlotinib-stimulated STAT3 phosphorylation in the tumor tissues (Figure 7).

## Discussion

Head and neck cancers are among the most prevalent tumors in the world. Despite advances in conventional therapies (i.e. surgery, radiation and chemotherapy), the overall survival rate for SCCHN has not significantly improved in the past three decades [4]. Therefore, the development of more effective new approaches that act through basic molecular mechanisms is critical to improve the prognosis of head and neck cancers. EGFR is widely overexpressed in SCCHN. Despite its ubiquitous expression, therapies targeting EGFR are only modestly effective in the treatment of SCCHN [5]. The mechanism of resistance to EGFR targeting agents is not fully understood. Here, we found that inhibition of EGFR by erlotinib results in phosphorylation of STAT3 at Tyr705 leading to its activation and to increased levels of Bcl2/Bcl-XL at both mRNA and protein levels in head and neck cancer cells (Figure 1). This effect could reduce the efficacy of erlotinib against head and neck cancer. Interestingly, erlotinib-induced STAT3 phosphorylation and activation did not result from its upstream kinase JAK2 because erlotinib does not activate JAK2, but contrarily reduces JAK2 phosphorylation (Figure 1A). In addition to JAK2, STAT3 phosphorylation is also tightly regulated by dephosphorylation through its physiologic phosphatase PTPMeg2 [15]. Treatment of Tu212 and Tu686 head and neck cancer cells with erlotinib reduced PTPMeg2 (i.e. a physiologic STAT3 phosphatase) in a time-dependent manner (Figure 1A). Thus, erlotinib, in addition to inhibition of EGFR, may also suppress PTPMeg2 leading to enhanced STAT3 phosphorylation. Intriguingly, specific knockdown of PTPMeg2 also activated the STAT3/Bcl2/Bcl-XL survival pathway (Figure 2), suggesting that PTPMeg2 may play an important role in regulating the sensitivity of erlotinib to head and neck cancer cells.

STAT3 has been recently identified as a potential therapeutic target for the treatment of head and neck cancer [37]. Depletion of STAT3 by RNAi significantly sensitizes head and neck cancer cells to erlotinib (Figure 3). Niclosamide has recently been identified as a STAT3 inhibitor that can suppress STAT3 phosphorylation at Tyr705 and transcript activity [18]. Since niclosamide not only potently blocks erlotinib-induced STAT3 phosphorylation but also synergizes with erlotinib against head and neck cancer *in vitro* and *in vivo* (Figures 4 and 5), we propose that erlotinib in combination with niclosamide has great potential to be developed as a new and more effective therapeutic approach for improving prognosis of head and neck cancer. Quantum dots (QDs) are nanoscale particles made from inorganic semiconductors and have novel optical properties that can produce fluorescence emission at different wavelengths depending on their size and composition [38–40]. The large stokes-shift of QDs, measured by the distance between the excitation and emission peaks, can be used to improve detection sensitivity. This is particularly important when analyzing complex biological samples such as tissue specimens which contain a high auto-fluorescence background and multiple biomarkers simultaneously. In addition, the QD-based immunohistofluorescence (QD-IHF) signal is not photobleachable and the signal spectrum is narrower than those from organic dyes, thereby enhancing specificity of the quantification. QD-based analysis confirmed that inhibition of pEGFR by erlotinib stimulates phosphorylation of STAT3 in tumor tissues, and that niclosamide specifically blocks erlotinib-induced STAT3 phosphorylation without affecting pEGFR (Figure 7). These QD-IHF data not only provide additional evidence that erlotinib activates STAT3, but also uncover the molecular mechanism by which niclosamide and erlotinib synergistically repress head and neck cancer *in vivo*.

In summary, the present studies have demonstrated that inhibition of EGFR by erlotinib stimulates phosphorylation and activation of STAT3 leading to increased levels of Bcl2 and Bcl-XL, which could reduce the efficacy of erlotinib against head and neck cancer. Erlotinib-induced activation of STAT3 may occur through suppression of its physiologic phosphatase PTPMeg2. Combined inhibition of EGFR and STAT3 using erlotinib and niclosamide synergistically represses head and neck cancer *in vitro* and *in vivo*, which may represent a novel and effective therapeutic strategy for improving prognosis of patients with head and neck cancer.

## References

[1] Sano D, Myers JN (2009) Xenograft models of head and neck cancers. Head Neck Oncol. 1: 32. 10.1186/1758-3284-1-32 PMC2737672 19678942

[2] Siegel R, Naishadham D, Jemal A (2013) Cancer statistics, 2013. CA Cancer J Clin 63: 11-30. doi:10.3322/caac.21166. PubMed: 23335087. 23335087 10.3322/caac.21166

[3] Leeman-Neill RJ, Cai Q, Joyce SC, Thomas SM, Bhola NE et al. (2010) Honokiol inhibits epidermal growth factor receptor signaling and enhances the antitumor effects of epidermal growth factor receptor inhibitors. Clin Cancer Res 16: 2571-2579. doi:10.1158/1078-0432.CCR-10-0333. PubMed: 20388852. 20388852 10.1158/1078-0432.CCR-10-0333 PMC2871379

[4] Forastiere A, Koch W, Trotti A, Sidransky D (2001) Head and neck cancer. N Engl J Med 345: 1890-1900. doi:10.1056/NEJMra001375. PubMed: 11756581. 11756581 10.1056/NEJMra001375

[5] Quesnelle KM, Wheeler SE, Ratay MK, Grandis JR (2012) Preclinical modeling of EGFR inhibitor resistance in head and neck cancer. Cancer Biol Ther 13: 935-945. doi:10.4161/cbt.20846. PubMed: 22785204. 22785204 10.4161/cbt.20846 PMC3414414

[6] Bollrath J, Phesse TJ, von Burstin VA, Putoczki T, Bennecke M et al. (2009) gp130-mediated. Stat 3 activation in enterocytes regulates cell survival and cell-cycle progression during colitis-associated tumorigenesis. Cancer Cell 15: 91-102 10.1016/j.ccr.2009.01.002 19185844

[7] Lee H, Deng J, Kujawski M, Yang C, Liu Y et al. (2010) STAT3-induced S1PR1 expression is crucial for persistent. Stat 3 activation in tumors. Nat Med 16: 1421-1428 10.1038/nm.2250 PMC3088498 21102457

[8] Lesina M, Kurkowski MU, Ludes K, Rose-John S, Treiber M et al. (2011). tat 3/Socs3 activation by IL-6 transsignaling promotes progression of pancreatic intraepithelial neoplasia and development of pancreatic cancer. Cancer Cell 19: 456-469

[9] Gao SP, Mark KG, Leslie K, Pao W, Motoi N et al. (2007) Mutations in the EGFR kinase domain mediate. Stat 3 activation via IL-6 production in human lung adenocarcinomas. J Clin Invest 117: 3846-3856 10.1172/JCI31871 PMC2096430 18060032

[10] Haura EB, Zheng Z, Song L, Cantor A, Bepler G (2005) Activated epidermal growth factor receptor-Stat-3 signaling promotes tumor survival in vivo in non-small cell lung cancer. Clin Cancer Res 11: 8288-8294. doi:10.1158/1078-0432.CCR-05-0827. PubMed: 16322287. 16322287 10.1158/1078-0432.CCR-05-0827

[11] Lai SY, Johnson FM (2010) Defining the role of the JAK-STAT pathway in head and neck and thoracic malignancies: implications for future therapeutic approaches. Drug Resist Update 13: 67-78. doi:10.1016/j.drup.2010.04.001. PubMed: 20471303. 10.1016/j.drup.2010.04.001 20471303

[12] Bharti AC, Donato N, Aggarwal BB (2003) Curcumin (diferuloylmethane) inhibits constitutive and IL-6-inducible. Stat 3 phosphorylation in human multiple myeloma cells. J Immunol 171: 3863-3871 10.4049/jimmunol.171.7.3863 14500688

[13] Inghirami G, Chiarle R, Simmons WJ, Piva R, Schlessinger K et al. (2005) New and old functions of STAT3: a pivotal target for individualized treatment of cancer. Cell Cycle 4: 1131-1133. doi:10.4161/cc.4.9.1985. PubMed: 16082218. 16082218 10.4161/cc.4.9.1985

[14] Yu H, Jove R (2004) The STATs of cancer--new molecular targets come of age. Nat Rev Cancer 4: 97-105. doi:10.1038/nrc1275. PubMed: 14964307. 14964307 10.1038/nrc1275

[15] Su F, Ren F, Rong Y, Wang Y, Geng Y et al. (2012) Protein tyrosine phosphatase Meg2 dephosphorylates signal transducer and activator of transcription 3 and suppresses tumor growth in breast cancer. Breast Cancer Res 14: R38. doi:10.1186/bcr3134. PubMed: 22394684. 22394684 10.1186/bcr3134 PMC3446372

[16] Xi S, Gooding WE, Grandis JR (2005) In vivo antitumor efficacy of STAT3 blockade using a transcription factor decoy approach: implications for cancer therapy. Oncogene 24: 970-979. doi:10.1038/sj.onc.1208316. PubMed: 15592503. 15592503 10.1038/sj.onc.1208316

[17] Boehm AL, Sen M, Seethala R, Gooding WE, Freilino M et al. (2008) Combined targeting of epidermal growth factor receptor, signal transducer and activator of transcription-3, and Bcl-X(L) enhances antitumor effects in squamous cell carcinoma of the head and neck. Mol Pharmacol 73: 1632-1642. doi:10.1124/mol.107.044636. PubMed: 18326051. 18326051 10.1124/mol.107.044636 PMC3437602

[18] Ren XM, Duan L, He QA, Zhang Z, Zhou Y et al. (2010) Identification of Niclosamide as a New Small-Molecule Inhibitor of the STAT3 Signaling Pathway. ACS Med Chem Lett 1: 454-459. doi:10.1021/ml100146z. 24900231 10.1021/ml100146z PMC4007964

[19] Chen Z, Zhang X, Li M, Wang Z, Wieand HS et al. (2004) Simultaneously targeting epidermal growth factor receptor tyrosine kinase and cyclooxygenase-2, an efficient approach to inhibition of squamous cell carcinoma of the head and neck. Clin Cancer Res 10: 5930-5939. doi:10.1158/1078-0432.CCR-03-0677. PubMed: 15355926. 15355926 10.1158/1078-0432.CCR-03-0677

[20] Ito T, Deng X, Carr B, May WS (1997) Bcl-2 phosphorylation required for anti-apoptosis function. J Biol Chem 272: 11671-11673. doi:10.1074/jbc.272.18.11671. PubMed: 9115213. 9115213 10.1074/jbc.272.18.11671

[21] Carbonaro M, Escuin D, O’Brate A, Thadani-Mulero M, Giannakakou P (2012) Microtubules regulate hypoxia-inducible factor-1alpha protein trafficking and activity: implications for taxane therapy. J Biol Chem 287: 11859-11869. doi:10.1074/jbc.M112.345587. PubMed: 22367210. 22367210 10.1074/jbc.M112.345587 PMC3320934

[22] Boisvert H, Duncan MJ (2010) Translocation of Porphyromonas gingivalis gingipain adhesin peptide A44 to host mitochondria prevents apoptosis. Infect Immun 78: 3616-3624. doi:10.1128/IAI.00187-10. PubMed: 20547744. 20547744 10.1128/IAI.00187-10 PMC2916282

[23] Huang S, Okumura K, Sinicrope FA (2009) BH3 mimetic obatoclax enhances TRAIL-mediated apoptosis in human pancreatic cancer cells. Clin Cancer Res 15: 150-159. doi:10.1158/1078-0432.CCR-08-1575. PubMed: 19118042. 19118042 10.1158/1078-0432.CCR-08-1575 PMC2948485

[24] Liu Y, Sun SY, Owonikoko TK, Sica GL, Curran WJ et al. (2012) Rapamycin induces Bad phosphorylation in association with its resistance to human lung cancer cells. Mol Cancer Ther 11: 45-56. doi:10.1158/1535-7163.MCT-11-0578. PubMed: 22057915. 22057915 10.1158/1535-7163.MCT-11-0578 PMC3256262

[25] Oltersdorf T, Elmore SW, Shoemaker AR, Armstrong RC, Augeri DJ et al. (2005) An inhibitor of Bcl-2 family proteins induces regression of solid tumours. Nature 435: 677-681. doi:10.1038/nature03579. PubMed: 15902208. 15902208 10.1038/nature03579

[26] Liu AW, Cai J, Zhao XL, Jiang TH, He TF et al. (2011) ShRNA-targeted MAP4K4 inhibits hepatocellular carcinoma growth. Clin Cancer Res 17: 710-720. doi:10.1158/1078-0432.CCR-10-0331. PubMed: 21196414. 21196414 10.1158/1078-0432.CCR-10-0331

[27] Xing Y, Chaudry Q, Shen C, Kong KY, Zhau HE et al. (2007) Bioconjugated quantum dots for multiplexed and quantitative immunohistochemistry. Nat Protoc 2: 1152-1165. doi:10.1038/nprot.2007.107. PubMed: 17546006. 17546006 10.1038/nprot.2007.107

[28] Xu J, Müller S, Nannapaneni S, Pan L, Wang Y et al. (2012) Comparison of quantum dot technology with conventional immunohistochemistry in examining aldehyde dehydrogenase 1A1 as a potential biomarker for lymph node metastasis of head and neck cancer. Eur J Cancer, 48: 1682–91. PubMed: 22341992. 22341992 10.1016/j.ejca.2011.12.029 PMC3381072

[29] Huang DH, Su L, Peng XH, Zhang H, Khuri FR et al. (2009) Quantum dot-based quantification revealed differences in subcellular localization of EGFR and E-cadherin between EGFR-TKI sensitive and insensitive cancer cells. Nanotechnology 20: 225102. doi:10.1088/0957-4484/20/22/225102. PubMed: 19433879. 19433879 10.1088/0957-4484/20/22/225102

[30] Wang X, Hawk N, Yue P, Kauh J, Ramalingam SS et al. (2008) Overcoming mTOR inhibition-induced paradoxical activation of survival signaling pathways enhances mTOR inhibitors’ anticancer efficacy. Cancer Biol Ther 7: 1952-1958. doi:10.4161/cbt.7.12.6944. PubMed: 18981735. 18981735 10.4161/cbt.7.12.6944 PMC2762753

[31] Chou TC (2010) Drug combination studies and their synergy quantification using the Chou-Talalay method. Cancer Res 70: 440-446. doi:10.1158/1538-7445.AM10-440. PubMed: 20068163. 20068163 10.1158/0008-5472.CAN-09-1947

[32] Fan W, Tang Z, Yin L, Morrison B, Hafez-Khayyata S et al. (2011) MET-independent lung cancer cells evading EGFR kinase inhibitors are therapeutically susceptible to BH3 mimetic agents. Cancer Res 71: 4494-4505. doi:10.1158/1538-7445.AM2011-4494. PubMed: 21555370. 21555370 10.1158/0008-5472.CAN-10-2668 PMC3132557

[33] Catlett-Falcone R, Landowski TH, Oshiro MM, Turkson J, Levitzki A et al. (1999) Constitutive activation of Stat3 signaling confers resistance to apoptosis in human U266 myeloma cells. Immunity 10: 105-115. doi:10.1016/S1074-7613(00)80011-4. PubMed: 10023775. 10023775 10.1016/s1074-7613(00)80011-4

[34] Bromberg JF, Wrzeszczynska MH, Devgan G, Zhao YX, Pestell RG et al. (1999). tat 3 as an oncogene. Cell 98: 295 10.1016/s0092-8674(00)81959-5 10458605

[35] Alas S, Bonavida B (2001) Rituximab inactivates signal transducer and activation of transcription 3 (STAT3) activity in B-non-Hodgkin’s lymphoma through inhibition of the interleukin 10 autocrine/paracrine loop and results in down-regulation of Bcl-2 and sensitization to cytotoxic drugs. Cancer Res 61: 5137-5144. PubMed: 11431352. 11431352

[36] Huang DH, Peng XH, Su L, Wang DS, Khuri FR et al. (2010) Comparison and Optimization of Multiplexed Quantum Dot-Based Immunohistofluorescence. Nano Research 3: 61-68. doi:10.1007/s12274-010-1009-1.

[37] Sen M, Joyce S, Panahandeh M, Li C, Thomas SM et al. (2012) Targeting. Stat 3 abrogates EGFR inhibitor resistance in cancer. Clin Cancer Res 18: 4986-4996

[38] Nie S, Xing Y, Kim GJ, Simons JW (2007) Nanotechnology applications in cancer. Annu Rev Biomed Eng 9: 257-288. doi:10.1146/annurev.bioeng.9.060906.152025. PubMed: 17439359. 17439359 10.1146/annurev.bioeng.9.060906.152025

[39] Bruchez M Jr., Moronne M, Gin P, Weiss S, Alivisatos AP (1998) Semiconductor nanocrystals as fluorescent biological labels. Science 281: 2013-2016. doi:10.1126/science.281.5385.2013. PubMed: 9748157. 9748157 10.1126/science.281.5385.2013

[40] Ferrari M (2005) Cancer nanotechnology: opportunities and challenges. Nat Rev Cancer 5: 161-171. doi:10.1038/nrc1566. PubMed: 15738981. 15738981 10.1038/nrc1566

